# The evolution of reproduction in Ediacaran–Cambrian metazoans

**DOI:** 10.1111/brv.70036

**Published:** 2025-05-15

**Authors:** Rachel A. Wood, Mary L. Droser

**Affiliations:** ^1^ School of Geosciences University of Edinburgh James Hutton Road Edinburgh EH9 3FE UK; ^2^ Department of Earth and Planetary Science University of California Riverside California 92521 USA

**Keywords:** Ediacaran, Cambrian, metazoan, reproduction

## Abstract

The evolution of reproductive style is a fundamental aspect of metazoan life history but has not been explored holistically through the Ediacaran–Cambrian rise of metazoans. Recent molecular clock analyses based on only unequivocal metazoan fossil calibrations suggest that Porifera were present by at least 590 million years ago (Ma), all major eumetazoan clades originated in the mid–late Ediacaran, and bilaterians were probably present by the late Ediacaran. An alternating pelagic larval (potentially for dispersal) and benthic adult life cycle appears to be an ancestral feature of metazoans. A compilation of inferred reproductive styles from the fossil record reveals that the low‐competition, deep‐water communities of the Ediacaran Avalon macrofossil assemblage (*ca*. 575 to 560 Ma) had current‐borne sexually produced larval with both local (non‐planktotrophic, with no feeding) and more widespread (planktotrophic, with feeding) dispersal followed by vegetative growth. By *ca*. 560 Ma, White Sea assemblage communities in shallow settings show dense aggregations, which were often dominated by single populations of episodic sexually produced larval spatfalls. Some taxa may show potential larval philopatry. By 550 Ma, with the rise of biomineralization and colonisation of shallow marine carbonate settings, the ability to encrust hard substrates, create multiple branches *via* budding, and rudimentary mutual attachment of inferred clones, first appear. The dominant apparent mode of reproduction throughout the Ediacaran was therefore *via* current‐borne sexually produced larvae followed by asexual reproduction, *via* either budding, fragmentation or fission. In these communities where biotic interactions were limited, this enabled colonisation of newly available soft and hard substrates followed by rapid growth. Early Cambrian communities showed increased endemism, enhanced trophic interactions and widespread macropredation. By the early Cambrian Fortunian stage (*ca*. 535 Ma), gonochorism (separate sexes) may have been present in priapulid worms. During Cambrian Stage 2 (*ca*. 532 Ma), internal fertilisation probably appeared in molluscs but widespread planktotrophy did not appear until the latest Cambrian/early Ordovician. Mutual attachment of diverse skeletal taxa became more common, particularly within reefs. Evidence for egg brooding and parental care in arthropods had appeared by the early Stage 3 (*ca*. 518 Ma). While reproductive styles were independently acquired, this overall pattern suggests a shift both to higher fecundity and to higher quality offspring in some groups during the Ediacaran–Cambrian Radiation, driven by increasing biotic interactions, including the rise of macropredation.

## I. INTRODUCTION: WHY REPRODUCTION MATTERS

How animals (metazoans) reproduce and invest resources in reproduction against incurred costs is a fundamental aspect of ecology and evolutionary biology (Subramoniam, 2018). While metazoans show a huge diversity of reproductive strategies, dynamic selective forces are involved as there is a universal trade‐off between offspring size and number (fecundity) (e.g. Smith & Fretwell, 1974). Overall fitness is determined by both the quality and quantity of offspring, but is limited by local resource availability. For example, in ecosystems with a high risk of predation or generally low survival, large numbers of smaller eggs are often produced at the cost of quality to offset initial mortality during larval (waterborne propagules) dispersal. By contrast, higher quality offspring tend to be lower in quantity, but are accompanied by greater investment in strategies that enhance survival such as protection from predation, parasitism, food shortages, or unfavourable environmental factors. How reproduction evolved in early metazoans therefore offers insight into evolutionary innovations and their interactions with changing environments and selective pressures (Strathmann, 1993). Many unrelated invertebrate taxa may share the same reproductive strategies, suggesting an independent evolutionary origin due to conserved developmental patterning systems and shared evolutionary pressures and constraints (Dani & Kodandaramaiah, 2017).

Modern large sessile invertebrates typically reproduce by means of larvae produced by sexual reproduction that are used for dispersal, followed by asexual reproduction of the adult phase usually *via* various types of vegetative growth when part of the benthos (Table 1). This is considered to be the ancestral state in metazoans (De Robertis & Tejeda‐Munoz, 2022). Larvae may be broadcast widely, or show only local dispersal with brooding dispersal, shown by aggregating behaviour. Philopatry describes a distribution pattern in which offspring remain close to their site of origin. Parental care is a widespread life‐history trait among animal groups that enhances protection from predators. It may encompass embryo protection and other complex behaviours such as brooding and carrying of pre‐hatched eggs by adult females, and is especially common in arthropods.

**Table 1:** Distribution of reproductive modes in modern large sessile invertebrates.

Reproductive mode				Phyletic distribution	Characteristics in fossil Record	Ecological significance
Asexual agamatic (clonal)	—	—	—	—	—	Potential for large numbers of offspring; rapid colonisation of new substrate; potential specialisation to habitats
	Vegetative growth	1. Budding (colonial and non‐ colonial)	—	Placozoa, Ctenophora, Cnidaria, Xenacoelomorpha, Bryozoa	Branching; buds; colonies/modular; stolons/runners	Colonise new, local, areas of substrate followed by rapid growth
	—	2. Fragmentation	—	Ctenophora, Cnidaria, Bryozoa, Nemertea, Xenacoelomorpha, Echinodermata, Hemicordata	Regrowth after breakage; multi‐generational clustering; Echinodermata: fissiparity (division of the body across the disk)	Rapid regrowth after storms/partial predation
	—	3. Fission	—	Placozoa	Multi‐size clustering on single bedding planes	—
	—	—	(a) Simple (binary)	Cnidaria, Echinodermata		—
	—	—	(b) Transverse	Cnidaria, Xenacoelomorpha, Platyhelminthes	Strobilation	—
	—	—	(c) Longitudinal	Annelida, Aceolomorpha, Platyhelminthes	Longitudinal fission	—
	—	Paratomy (ameiotic division)	—	Platyhelminthes	—	—
	Polyembryony (embryonic cloning)	—	—	Cnidaria, Bryozoans, Platyhelminthes, Arthropoda, Echinodermata	—	—
	Parthenogenesis (production of unfertilized eggs)	—	—	Platyhelminthes, Arthropoda, Rotifera	—	—
	Gemmule formation	—	—	Porifera	—	—
	Totipotency (stem‐cell regeneration)	—	—	Porifera	—	—
Sexual	—	—	—		—	Genetic variation; species can adapt to new environments to give survival advantage
	External fertilization: larvae	Broadcasting (long‐lived larvae + external fertilization)	—	Cnidaria, polychaetes, echinoderms	Wide dispersal	—
	—	Brooding (internal fertilization)	—	Cnidaria	Very local dispersal (endemic) + philopatry; clustered aggreations of same size individuals	Rapid substrate colonisation; reduction of competition from other clones
	Internal fertilization	—	—	Arthropoda, Mollusca, Annelida	—	—
	Parental care	—	—	Arthropoda, Mollusca	Nests: adult + egg clutch	Long juvenile stage; family bonding

The strategy of long‐lived waterborne larval stages capable of spending extended periods feeding in the plankton is known as planktotrophy. By contrast, the strategy of smaller, short‐lived larvae that are often negatively buoyant, have short dispersal distances, and rely solely on maternal reserves, is known as lecithotrophy. Both planktotrophic and lecithotrophic modes can be independently acquired. Lecithotrophic species have smaller population sizes, less gene flow (Shanks, 2009), and undergo more frequent extinctions compared to their planktotrophic relatives (Valentine & Jablonski, 1986). In modern climates, high latitudes and low temperatures favour lecithotrophy while tropical waters have more planktotrophic species, but many other local factors such as food availability and predation rates also favour one strategy over another (Zakas, 2022). The influences of all these controls during the Ediacaran–Cambrian are wholly unknown.

Gonochorism (or dioecism) whereby the sexes are always separate, as well as hermaphroditism, has evolved independently many times but is considered to be the ancestral (plesiomorphic) sexual mode in many clades. Most extant animals have separate sexes, but simultaneous hermaphrodites are also present throughout the Metazoa. Some phylogenetic analyses have shown that the transition from gonochorism to hermaphroditism has been more common in metazoan history than the reverse, except within the Bilateria (Sasson & Ryan, 2017). This suggests that factors that promote hermaphroditism in gonochoristic animals (e.g. reproductive assurance) are either more widespread or create stronger selective pressures than the conditions promoting gonochorism in hermaphroditic systems (e.g. inbreeding depression).

Metazoans can also show either internal or external fertilisation, and this can in turn affect fecundity. For example, the mean fecundity of gastropods with external fertilisation and planktic development is substantially higher than for gastropods with planktic development and internal fertilisation (Chaffee & Lindberg, 1986).

While many taxa reproduce seasonally, others are aseasonal, known as “continuous reproduction”, where multiple ages (and therefore body sizes) are found within communities. Modern benthic invertebrates (both as a whole and within specific taxonomic groups) in deeper‐water settings reproduce both seasonally and aseasonally; distinguishing between biological (i.e. continuous reproductive strategies) and environmental (lack of a seasonal trigger) causes for this pattern can be difficult.

The evolution of reproductive style has not been synthesised through the Ediacaran–Cambrian rise of metazoans, even though this is a critical part of understanding the rise of early metazoans to ecological dominance. Here we document the styles of inferred metazoan reproduction through the oldest putative fossil record of metazoans from the Ediacaran–early Cambrian interval, *ca*. 575–510 million years ago (Ma). This incorporates the Ediacaran Biota comprising the Avalon, White Sea and Nama assemblages, and the early Cambrian skeletal and soft‐bodied biota.

## II. INSIGHTS FROM DEVEOPMENTAL BIOLOGY

*Urbilateria*, the proposed last common ancestor of the protostome and deuterostome bilateral metazoans, was a complex animal, which possessed a *Hox* gene complex and many important structures already present such as eyes, a contractile circulatory system, and perhaps appendages (Butts, Holland & Ferrier, 2008). *Urbilateria* is inferred to have had a pelago‐benthic life cycle, where larvae had two rows of cilia beating in opposite directions to entrap food particles, an apical sensory organ, and a rudimentary eye. Acting on this conserved developmental patterning system, although channeled by deep homologies, selection allowed the evolution of diverse functions and morphologies (De Robertis & Tejeda‐Munoz, 2022). While the variety of morphologies found in modern marine larvae has evolved in response to serve different modes of movement and feeding, the ciliated trochophore is considered to be the ancestral form, and was present in *Urbilateria*.

While some have proposed that planktotrophy is the ancestral state of metazoans (Strathmann, 1985, 1993), molecular evidence suggests rather that lecithotrophy is the ancestral state (Peterson, 2005). Others have argued that direct development is ancestral, and larvae arose by co‐option of bilaterian adult‐expressed genes into independently evolved larval forms, where larvae may show morphological convergence but with distinct patterning genes (Raff, 2008). An ancestral lecithotrophic state supports the idea that the inferred predator‐free pelagic realm was first colonised by lecithotrophic larvae that evolved independently and multiple times during the Ediacaran to Early Cambrian, with the subsequent convergent evolution of planktotrophy from lecithotrophic ancestors at least four, and possibly up to eight, times during the late Cambrian to Middle Ordovician (Peterson, 2005). The rise of benthic suspension feeding is suggested to have driven increased fecundity, resulting in turn in selection for larval planktotrophy (Peterson, 2005; Signor & Vermeij, 1994). In this scenario, then, the increased fecundity conferred by planktotrophy would not be an adaptation to counter planktic mortality, but rather to counter mortality during settlement on the predatory benthos, as planktotrophic eggs are negatively buoyant (Peterson, 2005).

Most early Cambrian animals probably dispersed with non‐planktotrophic larval stages, whose appearance may have been driven by benthic predation pressures on eggs and developing embryos (Peterson, 2005). This would limit their geographic distribution. Benthic predation may also have driven multiple reversals back to lecithotrophy: although the loss of planktotrophy results in decreased fecundity, egg mortality may be lower as only juveniles rather than larvae would encounter the benthos (Peterson, 2005).

Larval pelagic and adult benthic metazoan life cycles have been proposed to have evolved at least four times independently during the late Ediacaran, where metazoans produced non‐feeding larvae (Signor & Vermeij, 1994). These four larval forms are presumed to be primitively epibenthic and non‐feeding (Haszprunar, Salvini‐Plawen & Rieger, 1995) and evolved independently from one another (Peterson, 2005): (*i*) the ciliated planula, found only in cnidarians; (*ii*) the ciliated trochophore in molluscs, annelids, and nemerteans; (*iii*) the ciliated dipleurula in echinoderms and hemichordates, and (*iv*) a non‐ciliated larval form, the tadpole, in ascidians.

## III. ASSESSMENT OF REPRODUCTIVE MODE IN THE FOSSIL RECORD

The reproductive characters of modern taxa can be inferred based on systematic affinity (assuming taxonomic uniformitarianism), biogeographic distribution, individual morphological/anatomical features and ecological/spatial distribution characteristics (Table 1). Many early metazoans lack direct evidence of reproductive style as reproductive organs have not been identified, so first we discuss how these might be inferred from the Ediacaran–Cambrian metazoan fossil record below, together with their attendant uncertainties.

The most profound issues facing analysis of reproductive styles in Ediacaran metazoans is the near‐complete absence of preserved inferred reproductive organs, and uncertainty as to systematic affinity: very few taxa have found a secure placement within modern phylogenetic trees because so few informative morphological characters have been recognised (but see, e.g. Dunn *et al*., 2021, 2022). In addition, the unusual preservational processes that produce Ediacaran soft‐bodied fossils within coarse, sandstone host rocks remain uncertain and disputed (e.g. Slagter *et al*., 2022), such that differentiating between features that are anatomical/morphological and those which are artefacts remains highly problematic.

### (1) Phylogenetic inference

The fossil record is biased by many factors, including an uneven stratigraphic record, human sampling bias, and differential preservation potential. Body fossils usually record clades which have reached a certain abundance and have a sufficiently distinct morphology to allow phylogenetic placement, and these will always postdate the genetic divergence of lineages (Carlisle *et al*., 2024).

**Fig. 1:**
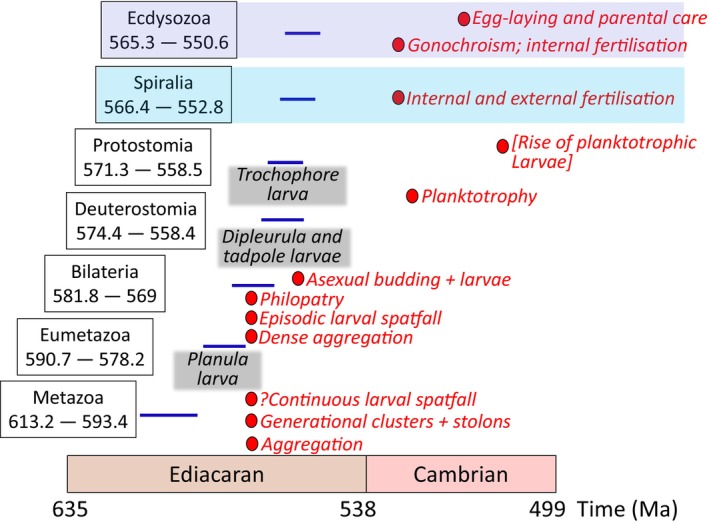
The appearance of major metazoan groups in millions of years based on the molecular clock analysis of Carlisle *et al*. (2024) (divergence date estimates together with errors in boxes and shown in blue lines) with inferred first appearance of non‐feeding larval types (in black), and first appearance of reproductive strategies found in the fossil record (in red) during the Ediacaran–Cambrian rise of metazoans, 575–510 million years ago (Ma).

The origin of the Metazoa has been suggested to be either Cryogenian or Tonian (e.g. Dos Reis *et al*., 2015), but a recent molecular clock analysis that incorporates only unequivocal metazoan fossils, revised and more precise fossil calibrations, and newly designated Ediacaran crown‐metazoan fossils, has yielded much younger divergence time estimates, with crown‐Metazoa originating in the early Ediacaran (an age estimate averaged across all analyses of 613.2 to 593.4 Ma; Carlisle *et al*., 2024). The earliest evidence for crown Bilateria is equivocal, but Bilateria must have diversified prior to the earliest Cambrian, as scalidophoran (Zhang *et al*., 2015), mollusc and chaetognath fossils (Steiner *et al*., 2007) have been described from strata of that age. A possible ecdysozoan has been described from strata of *ca*. 550 Ma (Hughes, Evans & Droser, 2024) and fossil data place total‐group euarthropod origination at or after ~550 Ma (Daley *et al*., 2018), similar to composites of molecular clock analyses which yield an estimate of 545.1 to 588.5 Ma (dos Reis *et al*., 2015). Broadly, these analyses suggest a radiation of metazoans beginning in the middle Ediacaran, with most crown‐phyla appearing during the late Ediacaran to early Cambrian and all major phyla originating by the late Cambrian, with the temporal gaps between the molecular clock clade age estimates and the fossil record minima being between 20 and 40 million years (Carlisle *et al*., 2024). On the basis of Carlisle *et al*. (2024), the estimated age for the first appearance of the four non‐feeding larval types is mid–late Ediacaran, with the planula larva appearing 590.7–578.2 Ma, dipleurula and the tadpole larvae by 574.4–558.4 Ma, and the trochophore larva by 566.4–552.8 Ma (Fig. 1).

### (2) Direct or inferred morphological fossil evidence

**Fig. 2:**
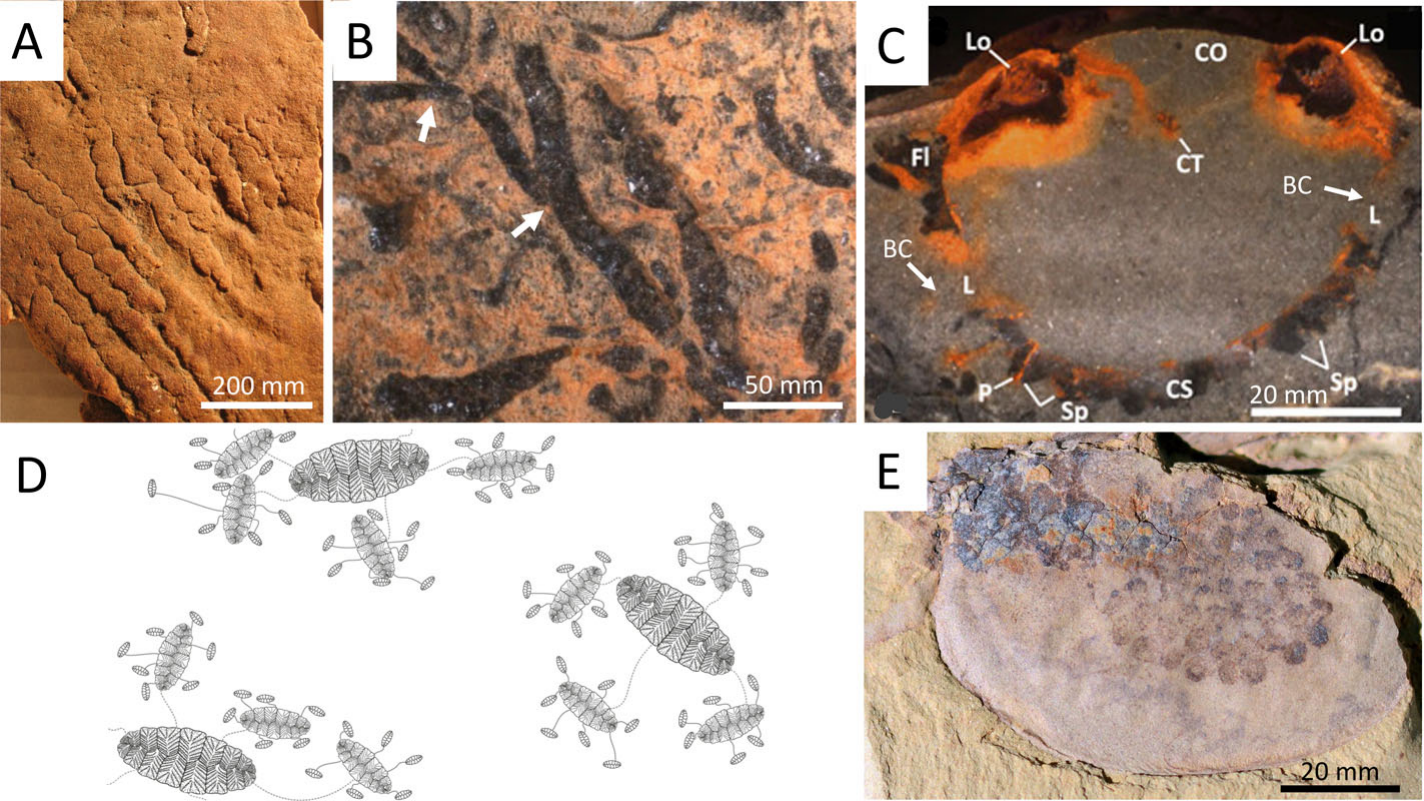
Examples of reproductive style in Ediacaran–Cambrian metazoans. (A) Clusters of *Funisia*, inferred to indicate philopatry, Ediacaran Member, Rawnsley Quartzite, Nilpena Ediacaran National Park, Australia, Ediacaran *ca*. 560 million years ago (Ma). Photograph credit: Mary Droser. (B) External budding (arrowed) in the skeletal tubular taxon, *Cloudina*, Nama Group, Namibia, Ediacaran *ca*. 547 Ma. Photograph credit: Amy Shore. (C) Cross section through *Namacalathus* with pyritised soft‐tissue, showing possible brood structures. BC, brood chamber (arrowed); CO, Central opening; CT, central structure; CS, calcite skeleton; Fl, flange; L, lumen; Lo, lobe; P, pore; Sp, spine. Nama Group, Namibia, *ca*. 547 Ma. Photograph credit: Amy Shore. (D) Schematic diagram showing simplified *Fractofusus* spatial arrangements, Mistaken Point, Newfoundland, Ediacaran *ca*. 575 Ma. Reproduced from Mitchell *et al*. (2015), *Nature* 524, 343–346, Fig. 4; Springer Nature. (E) Egg cluster in an arthropod. Laterally compressed specimen (ELEL‐SJ081254A) of *Chuandianella ovata* showing a cluster of eggs under its left carapace valve, early Cambrian Stage 3, *ca*. 520 Ma. Photograph credit: Qiang Ou, China University of Geosciences, Beijing (from Ou *et al*., 2020, *Sci. Adv*., 6, 3376–3405; AAAS).

In some cases of exceptional preservation, mainly in Lagerstätte, direct evidence for reproductive style can be preserved in body fossils. This includes egg clusters (e.g. Duan *et al*., 2014; Hegna, Martin & Darroch, 2017; Ou *et al*., 2020; see Fig. 2E), and possible reproductive structures (e.g. Jenkins, 1992; Shore *et al*., 2021).

Individual fossil evidence for vegetative growth can be inferred from a modular, clonal organisation (e.g. *Namapoikia*; Wood, Grotzinger & Dickson, 2002), or various budding organisations (e.g. see *Cloudina*, see Fig. 2B). Other macrofossil features have also been suggested to be associated with reproduction. Extensive, linear structures have been inferred to be stolons in Avalon rangeomorph taxa (Liu & Dunn, 2020; Mitchell *et al*., 2015; see Fig. 2D), and inference has also made *via* phyletic analogy with modern taxa. It has been speculated that the bullae (bulbous structures at the midpoint of each curving arm) on the White Sea soft‐bodied taxon *Tribrachidium* may represent reproductive structures (Jenkins, 1992), although this has not been supported by the application of computational fluid dynamics (Olaru *et al*., 2024; see below). Preservation of inferred soft tissue *via* pyritization across the (generally six) lumens of the skeletal taxon *Namacalathus* has been suggested to correspond to brood chambers formed by external body wall invaginations, as found in bryozoans (Shore *et al*., 2021; see Fig. 2C).

Overall adult shell morphology and size, and protoconch size, in univalved molluscs indicates fecundity and larval type, so can be used to construct models that estimate Cambrian univalve reproductive style (Chaffee & Lindberg, 1986; Nützel, Liehnert & Frýda, 2006). A large protoconch (230–330 μm in width), indicates lecithotrophic larval development, but a small two‐stage protoconch (60–120 μm) is produced by taxa with planktotrophic larvae. The relatively small body size of most Early Cambrian molluscs infers potential fecundities substantially below those correlated with planktic development in Recent gastropods, and so suggests a non‐planktic mode of larval development, with external fertilisation, relatively large eggs, and lecithotrophic development as the ancestral condition. The larger body size found in Late Cambrian and Early Ordovician molluscs suggests increased fecundities similar to those correlated with planktotrophic development. Chaffee & Lindberg (1986) therefore proposed that the evolution of larval development in Cambrian molluscs proceeded from non‐planktic lecithotrophic development to planktic lecithotrophic development and that planktotrophic larvae are secondarily derived. The increased sizes of the Mollusca in the late Cambrian and early Ordovician (Runnegar, 1983) may have been a response to increase fecundity (Chaffee & Lindberg, 1986), which coincided with planktotrophic larval development as part of the Ordovician Radiation, perhaps in turn related to the rise of benthic suspension‐feeding (Nützel *et al*., 2006; Nützel, 2014; Runnegar, 2007; Signor & Vermeij, 1994).

### (3) Biogeographic distribution

The global *versus* regional *versus* endemic biogeographic distribution of some groups can be interpreted as evidence for reproduction *via* waterborne larvae or other propagules. Two problematic issues arise, however, first with inadequate study of potential taxonomic *versus* ecophenotypic variability of Ediacaran macrofossils, and the second with biases in the Ediacaran fossil record.

Many Ediacaran taxa have been studied in geographic isolation and without the taxonomic scrutiny afforded by more abundant fossils, such that supposed distinct or endemic species may in fact be ecophenotypes (e.g. Wood *et al*., 2017). Many Ediacaran soft‐bodied taxa are also noteworthy for being single‐species genera.

Analyses of biogeographic distributions requires large data sets to reach statistical significance. But the diversity (species richness) of all Ediacaran metazoans in the Avalon, White Sea and Nama assemblages has been shown to be significantly under‐sampled, with reconstructed diversity more a consequence of highly patchy sampling intensity and the uneven distribution in space and time of hosting sedimentary rocks with notable palaeolatitudinal and depositional setting biases (Bowyer, Wood & Yilales, 2024). This means that some claims about changing dispersal patterns through the Ediacaran may be statistically unfounded.

A statistically significant change in co‐occurrence patterns of genus pairs, an increase in depth structure, and more niche specialisation, has been noted between each Ediacaran assemblage, particularly between the White Sea and Nama assemblages, and this is inferred to indicate complex patterns of ecological and/or biogeographic change (Caffrey *et al*., 2024; Eden, Manica & Mitchell, 2022). But even when estimated genus richness confirms biota differences (Muscente *et al*., 2019), the sampling intensity, depth ranges, and geographic coverage of these assemblages are strikingly different, with the Nama Assemblage being notably under‐sampled, especially as few bedding plane communities have been documented compared with the Avalon and White Sea assemblages (Bowyer *et al*., 2024).

### (4) Ecological and statistical distribution within communities

Inference of underlying ecological processes can be gained *via* their recurrent, statistical spatial and size‐frequency distributions on bedding planes that represent the colonised sea floor (e.g. see *Fractofusus*, Fig. 2D). Taxa with seasonal reproduction and recruitment typically show distinct modes in size frequency (e.g. Fujita & Ohta, 1990), and spatial point process analyses can detect increased densities in areas of higher‐quality habitat within patchy, heterogenous settings (Baddeley & Turner, 2005). Such increased densities or aggregations can be statistically distinguished from other aggregation processes that are related to dispersal limitation and reproductive processes. Larval spatfalls may represent single or multiple generations, and larval spatfall is most likely to be current‐borne and sexually produced, and so will enable rapid colonisation of new substrate. Philopatry can be demonstrated by a pattern of offspring remaining by their birthplace, but lecithotrophy can only be inferred from this dispersal limitation (e.g. see *Funisia* in Fig. 2A).

### (5) Experimental approaches

Decay experiments suggest that the most labile tissues might be preferentially lost in soft‐bodied Ediacaran taxa, further increasing bias against preservation of key morphological features (Gibson, Sciffbauer & Darroch, 2018). For example, a critical argument against the Ediacaran White Sea taxa *Kimberella* being a mollusc is the lack of a preserved hemocoel (Erwin, 1999), but it is precisely this key feature which might be lost rapidly during decay.

Computational Fluid Dynamics (CFD) is being increasingly used to test feeding or reproductive style hypotheses. For example, studies using CFD modelling have supported the hypothesis that the Nama assemblage taxon *Ernietta* likely were gregarious suspension feeders (Gibson *et al*., 2019). CFD was used to test the origin of linear arrangements of closely spaced fossil rangeomorphs on Avalon‐aged surface (known as “conga lines”), where the authors concluded that such an organisation was unlikely a result of preferential leeside settlement but rather revealed a reproductive arrangement following stolons (Delahooke *et al*., 2024). CFD analysis also showed that proposed reproductive structures (bullae) on the White Sea soft‐bodied taxon *Tribrachidium* (Jenkins, 1992) did not induce vertical flow that would support a function that facilitated wider gamete release into the water column (Olaru *et al*., 2024).

## IV. THE RECORD OF EDIACARAN–CAMBRIAN REPRODUCTIVE STYLES

In Table 2, we assemble available data on the distribution of inferred reproductive styles in fossil metazoans from the Ediacaran interval (*ca*. 575–538 Ma) from the literature together with new field observations.

**Table 2:** Distribution of inferred reproductive styles in fossil metazoans from the Ediacaran, *ca*. 575–538 million years ago (Ma).

Taxon	Age (ma)	Affinity	Reproductive mode	Local spatial distribution	Palaeobiogeography	Reproductive style	Reference
*Cloudina*	*ca*. 550–536	?	Internal budding	Aggregation	Global	Asexual – budding Possible episodic larval spatfalls, accompanied by larval philopatry?	Hua *et al*. (2005); Penny *et al*. (2014)
Cloudinomorphs	*ca*. 550–536	?	External budding	Aggregation	Global	Asexual – budding; sexual – broadcast larvae?	Cortijo *et al*. (2010)
	—	—	—	—	—	Asexual – budding; possible episodic larval spatfalls, accompanied by larval philopatry?	—
*Namacalathus*	*ca*. 550–540	?Lophophorate	External budding	Aggregation	Widespread	Possible brood chambers formed by external body wall invaginations	Penny *et al*. (2017); Shore *et al*. (2021)
*Corumbella*	*ca*. 540	?	External budding	Aggregation	Laurentia	Asexual – budding	Pacheco *et al*. (2015)
*Namapoikia*	*ca*. 547	?Poriferan	Clonal/colonial?	Encruster; cryptic	Endemic	Totipotency	Wood *et al*. (2002)
*Ernietta*	*ca*. 550–536	?Cnidarian		Aggregation	Global	*In‐situ* adult aggregations may have created localised condition to aid settlement and growth of younger individuals	Gibson *et al*. (2021)
*Coronacollina*	*ca*. 560–540	?	—	—	Endemic	—	—
*Sinotubulities*	*ca*. 550–540	?	—	—	?Endemic	—	—
*Arborea*	573–540	?	—	Aggregation	Global	—	Peterson *et al*. (2003)
*White Sea* *Assemblage, Russia*	*ca*. 560–540	—	—	Multiple size modes		Seasonal colonisation of the sea floor by larvae	Zakrevskaya (2014)
*Funisia*	*ca*. 560–540	?Cnidarian	—	Aggregation; high dominance	Endemic	Sexually produced spatfalls potentially indicating larval philopatry; asexual – budding?	Droser & Gehling (2008)
*Tribrachidium*	*ca*. 560–540	?	—	Random or clustered; high dominance	Global	?Seasonal spatfall	Hall *et al*. (2015)
*Dickinsonia*	*ca*. 560–540	Eumetazoa	—	Random	Global	?Sexual larvae	Xiao *et al*. (2013); Droser *et al.(* 2019); Evans *et al*. (2022)
*Obamus*	*ca*. 560–540	?	—	Aggregation	Endemic	—	—
*Spriggina*	*ca*. 560–540	?	—	Random	Endemic	?Sexual larvae; small body sizes concentrated	Xiao *et al*. (2013); Droser *et al*. (2019)
*Andiva*	*ca*. 560–540	Eumetazoa	—	Random	Global	?Sexual larvae	Evans *et al*. (2020)
*Rugoconites*	*ca*. 560–540	?	—	Clustered or random; can be high dominance	Global	?Seasonal spatfall	Hall *et al*. (2018)
*Plexus*	*ca*. 560–540	?	—	Clustered; high dominance	Endemic	—	Joel *et al*. (2014)
*Parvancorina*	*ca*. 560–540	?	—	Juveniles can be clustered	Global	Sexual reproduction	Coutts *et al*. (2018)
*Kimberella*	*ca*. 560–540	?Stem‐group mollusc	—	Random	Global	?Sexual larvae	Fedonkin *et al*. (2007)
Rangeomorphs	*ca*. 573	?	—	—	—	Inferred stolons	Liu & Dunn (2020); Delahooke *et al*. (2024)
			—	Size distribution of juveniles	—	Continuous reproduction model (possibly seasonal)	Liu *et al*. (2012)
			—	Wide variance in size with right‐skewed size‐frequency distributions	Global	Planktotrophic larvae; continuous reproduction model (possibly aseasonal)	Darroch *et al*. (2013)
*Fractofusus*	*ca*. 573	?	—	—	—	Single cohort with wide variance; continuous reproduction model (possibly aseasonal)	Mitchell *et al*. (2015)
	—	—	—	—	—	Inferred stolons	Fedonkin (1985); Peterson *et al*. (2003); Mitchell *et al*. (2015)
	—	—	—	—	—	Multigenerational asexual clonal phase, interspersed with the release of waterborne propagules; generational clusters; inferred stolons; rapid exploitation of localised areas, as well as transport to new, previously uncolonised areas	Mitchell *et al*. (2015)

The oldest macrobiota communities, known as the Avalon assemblage (*ca*. 575–546 Ma) are dominated by a soft‐bodied sessile benthos of frondose organisms known as rangeomorphs. These are assumed to have been suspension‐feeders on water column plankton and particulate organic matter/particulate organic carbon (Butterfield, 2022). Statistical analyses suggest that there was limited evidence of competition for resources, with only rare intra‐ and interspecific competition (see review in Mitchell & Pates, 2025). Studies have found that the majority of Avalon assemblage biota had shared niches, so likely shared food sources (Darroch, Laflamme & Wagner, 2018). Macropredation appears to have been absent, and hence environmental and resource controls (which may not have been limiting) rather than biotic interactions have been suggested to have been dominant in community structuring (Mitchell & Pates, 2025).

Levels of endemism are generally (but not exclusively) extremely low for much of the Ediacaran (Muscente *et al*., 2019), and many taxa were able to cross oceanic basins to achieve an inferred cosmopolitan distribution (Darroch, Laflamme & Clapham, 2013). This suggests widespread planktotrophy, although endemism is suggested to be higher for the White Sea than the Avalon assemblage (Eden *et al*., 2022). A number of reproductive strategies have been proposed for taxa of the deep‐water Ediacaran Biota Avalon assemblage (ca. 575–546 Ma), but all may have shared a planktonic (but not necessarily planktotrophic) early stage prior to development on the sediment substrate which suggests sexual reproduction by means of gametogenesis (Table 2). The global distribution of charniids (from the rangeomorph sub‐group) has been interpreted as evidence for planktotrophic larvae (Darroch *et al*., 2013). Likewise, *Arborea* is one of the most abundant Ediacaran taxa known from all three Ediacaran assemblages and on multiple continents. Some rangeomorph assemblages show a wide variance in size with right‐skewed size‐frequency distributions, inferred to indicate continuous aseasonal sexual reproduction, although a similar distribution could be due to seasonal reproduction combined with slow growth rates (Darroch *et al*., 2013). While spatfall may be present, continuous larval spatfall has not been confirmed and cannot be inferred from size distributions alone.

Assemblages of other diverse juvenile rangeomorph taxa suggests that reproduction may also have been seasonal (Liu *et al*., 2012). The presence of multi‐generation clusters in *Fractofusus* has been used to infer stolon‐like (specialised outgrowths that connect parent to offspring) asexual clonal reproduction (Mitchell *et al*., 2015; Fig. 2D), supported by the presence of linear structures interpreted as potential filamentous stolons amongst diverse rangeomorph taxa (Liu & Dunn, 2020). Among those taxa which are associated with stolon‐like structures, some, such as *Fractofusus* are endemic and others such as *Charnia*, are more widespread, but such clonal reproduction was likely combined with dispersal by sexual waterborne propagules. “Conga lines” have been described as linear arrangements of more than three closely spaced fossil rangeomorphs on Avalon‐aged surface (Delahooke *et al*., 2024). Using CFD, the authors concluded that such an organisation was unlikely a result of random chance or preferential leeside settlement and they suggested the use of both dispersal‐limited and propagule reproductive strategies in the earliest animals enabled mixed, plastic, and/or generalist reproductive strategies in rangeomorphs. Different Avalon assemblage taxa may have thus had different dispersal abilities.

Beta diversity (a measure of how much variation is present between communities) is significantly higher in Ediacaran communities than in the Cambrian and later. Strong dispersal limitation, created *via* asexual stoloniferous reproduction (Mitchell *et al*., 2015), may explain this patchiness.

The White Sea assemblage (*ca*. 560–540 Ma) represents an increase in taxonomic and morphological diversity as well as in behaviour and ecology (e.g. Droser, Tarhan & Gehling, 2017; Xiao & Laflamme, 2009; Table 2). The extent of intra‐ and interspecific competition is thought to have increased (Droser *et al*., 2017), in tandem with an increase in the relative importance of niche/environment interactions and their complexity (Droser *et al*., 2022). Several taxa have a global record including *Dickinsonia*, *Arborea*, *Parvancorina*, *Tribrachidium*, *Kimberella* and *Andiva*, potentially indicating an ability to disperse over great distances. Given the patchy nature of our current record, however (see Bowyer *et al*., 2024), the attribution of a global distribution to these genera and not others may be a function of either increased sampling and/or higher global abundance.

Many White Sea taxa are inferred to have been capable of sexual reproduction, for example *Dickinsonia* (Fedonkin, Simonetta & Ivantsov, 2007). White Sea assemblage communities in shallow settings show patchy but dense aggregations that appear to be indicative of the likely sexually produced spatfalls potentially indicating larval philopatry, for example *Funisia* (Fig. 2A). These dense communities show high community heterogeneity between environments (Droser *et al*., 2019), and are dominated by populations with a very limited individual size range (e.g. for *Tribrachidium* and *Rugonconites*) suggesting episodic larval spatfalls (Hall *et al*., 2015; Hall, Droser & Gehling, 2018; Boan *et al*., 2023). Similarly, using a statistical method of likelihood‐based model selection (Bayesian Information Criterion) and cluster analysis allowed the inference of multiple size modes of White Sea fossil assemblages reflecting seasonal colonisation of the sea floor by larvae (Zakrevskaya, 2014). Preferential settlement has been noted on patchy or variably stable substrates, leading to communities of differing maturity composition (Droser *et al*., 2022).

The globally distributed taxon *Parvancorina* typically occurs as individual specimens, suggesting efficient dispersal, but some aggregate with a bimodal distribution, particularly in smaller, inferred juvenile cohorts (Coutts *et al*., 2018). This pattern is interpreted, however, to be a response to benthic water currents rather than a product of reproduction, as small, assumed juvenile, specimens show a strong axial current alignment, in contrast to the unimodal orientations observed in larger specimens.

The five mobile White Sea taxa inferred to be of bilaterian grade (*Dickinsonia*, *Yorgia*, *Kimberella*, *Ikaria* and *Uncus*) left trace fossils that indicate efficient mobility associated in part with feeding. *Andiva*, *Spriggina* and *Parvancorina* were also likely to have been mobile (but the latter possibly only facultatively; Darroch *et al*., 2017), based on shared morphology and relationships to known mobile taxa, and there is no documentation of these taxa aggregating, overlapping or otherwise being in contact. But dense and abundant assemblages of *in situ* filamentous fossils interpreted as algae (Xiao *et al*., 2013) have been found on some surfaces at Nilpena Ediacaran National Park, Australia, associated with *Dickinsonia*, *Parvancorina* and sprigginamorphs, where the diameter of *Dickinsonia* individuals is <1 cm, significantly smaller than other contemporary individuals (Evans *et al*., 2020). Such an unusual fossil assemblage suggests an algal‐dominated community that served as a nursery for mobile macro‐taxa (*cf*. Liu *et al*., 2012; Xiao *et al*., 2013). Studies of modern ecosystems have noted preferential larval settlement in areas characterised by certain types of biofilm cover, where cues from biofilms may “inform” larvae that a particular biofilm is well developed and stable (Hadfield, 2011). Predation pressure is also commonly considered a strong driver of algal‐hosted nursery development, but no evidence for significant predation has been documented in Ediacaran ecosystems. Nonetheless, the presence of densely packed “thickets” of algae, coupled with biofilm development, may have provided a safe haven for larvae in otherwise high‐energy conditions.

Shallow carbonate depositional settings were first extensively colonised during the Nama Assemblage (*ca*. 550–535 Ma), occupied by new, sessile, skeletal metazoans that effectively opened a new taphonomic window that has continued ever since. Most forms have a clear preference for attachment to microbial mats or stromatolitic/thrombolitic substrates. *Cloudina* (a possible cnidarian or annelid) and *Namacalathus* (a possible lophotrochozoan) show dense aggregations of the same individual size, suggesting that communities are dominated by single populations of episodic larval spatfalls, possibly accompanied by larval philopatry (Penny *et al*., 2014, 2017; Wood *et al*., 2017). The first potential macroscopic predation is documented as drillholes in *Cloudina* (Bengston & Yue, 1992).

Several new reproductive strategies appear, including the ability to encrust hard substrates *via* inferred modular/clonal propagation (*Namapoikia*) (Wood *et al*., 2002), and possible bilateral budding (*Namacalathus*) (Zhuravlev, Wood & Penny, 2015), internal budding (cloudinomorphs) (Hua *et al*., 2005; Cortijo *et al*., 2010, 2014), and multiple branching *via* external budding (*Cloudina*; Fig. 2B) (Shore *et al*., 2020). Rudimentary mutual attachment of the same taxa, and possibly inferred clones, is also documented in aggregating *Cloudina* (Penny *et al*., 2014). Preservation of inferred soft tissue *via* pyritization across the (generally six) lumens of the skeletal taxon *Namacalathus* has been suggested to correspond to brood chambers formed by external body wall invaginations, as found in bryozoans (Shore *et al*., 2021; see Fig. 2C).

Relatively few studies have sought to infer the reproductive modes of soft‐bodied taxa from the Nama assemblage, mainly due to the fact that unlike the Avalon and White Sea assemblages, few *in‐situ* bedding plane communities have been described. An exception is the poorly dated carbonate Shibantan Lagerstätte, South China (*ca*. 551–*ca*. 543 Ma), which records diverse bedding‐plane assemblages including mobile bilaterian animals such as the large endemic segmented *Yilingia*, typical Ediacaran soft‐bodied suspension feeders, biomineralising taxa, macroalgae, and ichnotaxa (Xiao *et al*., 2021).

Studies using CFD modelling have supported the hypothesis that the Nama assemblage taxon *Ernietta* likely behaved as gregarious suspension feeders (Gibson *et al*., 2019), and that *in‐situ* aggregations of adults may have performed a “nursery” function, creating localised conditions ideal for the settlement and growth of younger individuals (Gibson *et al*., 2021).

If Nama assemblage taxa such as *Ernietta*, *Cloudina* and *Namacalathus* were suspension‐feeders, then they may signal the rise of planktotrophy (the capacity to feed upon eukaryotic/metazoan plankton) in the benthos (Wood & Curtis, 2015), although it has been argued that this capacity was not dominant until the Ordovician Radiation (Signor & Vermeij, 1994).

The Cambrian saw a notable increase in small shelly taxa with a diversity of mineralogies, as well as a rise in ichnotaxa and many lagerstätten that reveal diverse communities where taxa can be placed with modern phyla. Endemicity, provinciality and a reduction in geographic ranges, sediment mixing due to enhanced burrowing, and macropredation all increased during the early Cambrian (Erwin *et al*., 2011). With this came an inferred shift from low competition to high competition for resources and enhanced trophic interactions (Na & Kiessling, 2015).

The first unequivocal mollusc fossils are known from the Cambrian Fortunian. The relatively small body size of early Cambrian molluscs has been used to infer low fecundities, a non‐planktic mode of larval development, large eggs, and lecithotrophic development (Chaffee & Lindberg, 1986), with planktotrophic larvae only appearing in this group in the late Cambrian (Nützel *et al*., 2006; Nützel, 2014; Runnegar, 2007).

The first evidence for planktotrophy (the rapid appearance of antipredatory spines in planktic acritarchs), is found in Cambrian Stage 2 (Butterfield, 1997). During this interval, mutual attachment of different taxa of reef‐building skeletal metazoans (archaeocyath sponges, coralomorphs, and cribricyaths) became common. Female tubular paired gonads with a relatively low number (<30) of large size (compared to modern macrobenthic taxa) have been identified in selkirkiid priapulid worms in the exceptionally preserved material from the Cambrian Stage 3 Xiaoshiba Lagerstätte (Yang *et al*., 2021; 514 Ma), but priapulid worms were present by the latest Ediacaran or basal Cambrian, supported by both phylogenetic trees and molecular clock analysis (Howard *et al*., 2022; Carlisle *et al*., 2024) and trace fossils (Turk *et al*., 2024).

Ancestral arthropods are inferred to have released their gametes with subsequent external fertilisation, as inferred from the trilobite *Triarthrus* with associated eggs (Hegna *et al*., 2017). The evolution of copulation would have increased fertilisation success, and this would allow for increasing parental care (Ou *et al*., 2020; Fu *et al*., 2018). Brooding and egg‐carrying by adult females is evidenced by the fossilised eggs carried in the posterior appendages of *Kunmingella douvillei*, an ostracod‐like arthropod (Duan *et al*., 2014), and under the lateral flaps of the carapaces of *Chuandianella ovata* (e.g. Ou *et al*., 2020; Fig. 2E), both from the early Cambrian (Stage 3, *ca*. 520 Ma), Chengjiang biota. Fossilised embryos are known from *Waptia* spp., a “bivalve” arthropod from the middle Cambrian Burgess Shale (Caron & Vannier, 2016; Ou *et al*., 2020; *ca*. 510 Ma). These reveal that two distinct reproductive strategies were already present in waptiids: females of *C. ovata* brooded a relatively high number of small eggs, whereas those of *W. fieldensis* carried a low number of large eggs.

## V. DISCUSSION: THE RISE OF DIVERSE REPRODUCTIVE MODES

Both external fertilisation and the possession of pelagic larvae in many marine invertebrates are considered to be primitive features of reproduction, which confer a clear advantage for colonising new habitats. The predominance of vegetative reproductive and current‐borne sexually produced larvae indicates the co‐evolution of both asexual and sexual reproductive processes in exploiting environmental conditions. These dominant modes of reproduction throughout the Ediacaran enabled colonisation of newly available soft and hard substrates followed by rapid growth, in an environment of low competition and absent macropredation. This suggests a premium on high fecundity, perhaps further suggesting low‐quality offspring.

The wide range of new body plans that characterise the early Cambrian were accompanied by the rapid development of new feeding strategies and expansion of prey–predator relationships (Erwin *et al*., 2011). These new body plans were accompanied by new modes of reproduction, found to date in priapulids, molluscs and arthropods.

Priapulids can show both external and internal fertilisation, but the basic organisation of the reproductive system into paired ovaries and oviducts was probably established very early in the evolution of the group (Yang *et al*., 2021). In modern meiobenthic priapulids, enhanced offspring survivorship may be *via* the production of only a small number of yolk‐rich embryos (Yang *et al*., 2021). The fossil embryos and post‐embryonic stages of scalidophoran worms from the Fortunian also are of a relatively large size (Donoghue *et al*., 2006).

Brooding and egg‐carrying by adult females are frequent in modern pancrustacean groups, and this basic form of brood care appears to have arisen early in arthropod evolution. Copulation and internal fertilisation may also have a very ancient origin among euarthropods (Yang *et al*., 2021).

An increase in beta diversity, that is differentiation among communities or assemblages, occurs in tandem with increasing gamma diversity during the Cambrian radiation (Na & Keissling, 2015), with beta diversification showing the pattern predicted for a low‐competition scenario of unbounded diversification *via* niche‐partitioning potentially driven by predatory escalation. An alternative scenario is that the increase of geographic beta diversity during the early Cambrian might be driven by the widespread appearance of animals with larval stages combined with a notable phase of continental break‐up (Na & Keissling, 2015).

We can therefore construct a testable model of the timing of modes of reproduction through the early evolution of metazoans based on the fossil record (Fig. 1). The deep‐water communities *ca*. 575–560 Ma of the Ediacaran Avalon assemblage showed both local (non‐planktotrophic) and widespread (planktotrophic) larval dispersal followed by vegetative growth *via* stolons. By *ca*. 560 Ma, White Sea assemblage communities in shallow settings show dense aggregations, with some dominated by single populations of inferred episodic larval spatfalls, and others with potential larval philopatry. While these waterborne propagules were most likely larvae, it is not possible to exclude the presence of microscopic buds or fragments.

By 550 Ma, with the rise of biomineralisation, the ability to encrust hard substrates, create multiple branches *via* budding, and rudimentary mutual attachment of inferred clones, first appear. By the early Cambrian Fortunian, the first evidence for internal fertilisation, paired gonads and gonochorism, appears, by Stage 2 there is evidence for planktotrophy, and evidence for egg brooding and parental care in arthropods is present by the early Stage 3 Cambrian. Planktotrophy became increasingly widespread from the late Cambrian.

In sum, the dominant mode of reproduction throughout the Ediacaran was *via* sexually produced larvae and adult vegetative growth, which enabled rapid colonisation of newly available soft and hard substrates followed by fast adult growth. Philopatry can also be inferred to be present, and gonochorism widespread. Molecular clock predictions show that new reproductive modes (internal fertilisation, paired gonads, and parental care) had evolved at least by the late Ediacaran. These are confirmed by body fossils from the earliest Cambrian, and with this the ability to produce fewer, but higher quality, eggs and larvae.

## VI. CONCLUSIONS

(1) The first known Ediacaran metazoans (*ca*. 575–560 Ma), which formed deep‐water marine communities of the Avalon assemblage, showed both local (non‐planktotrophic, with no feeding) and widespread (planktotrophic, with feeding) current‐borne sexually produced larval dispersal followed by vegetative growth.
(2) The White Sea assemblage communities, which appeared *ca*. 560 Ma in shallow settings, show dense, often monospecific aggregations, which were often dominated by single populations of inferred episodic larval spatfalls, some with potential larval philopatry.
(3) By 550 Ma, with the rise of biomineralization, the ability to encrust hard substrates, create multiple branches *via* budding, and rudimentary mutual attachment of inferred clones, first appear.
(4) The documented dominant mode of reproduction throughout the Ediacaran was *via* asexual reproduction, whether *via* stolons, budding, fragmentation or fission, and current‐borne sexually produced larvae, both of which enabled colonisation of newly available soft and hard substrates followed by rapid growth. This reproductive mode may have been dominant given the low levels of competition, trophic interactions, and absence of macropredation.
(5) Although molecular clocks predict an earlier appearance, body fossils from the early Cambrian Fortunian stage (*ca*. 535 Ma) first document gonochorism (separate sexes), with internal fertilisation appearing in Cambrian Stage 2 (*ca*. 532 Ma), and egg brooding and parental care by the early Stage 3 (*ca*. 518 Ma). Planktotrophy became increasingly widespread from the late Cambrian.
(6) While reproductive styles were independently acquired, this overall pattern suggests a shift both to higher fecundity and to higher quality offspring in some groups during the Ediacaran–Cambrian Radiation. This was probably driven by increasing biotic interactions, including substrate competition and the rise of micropredation.
